# Evaluating pharmacy students’ knowledge and skills in reproductive, maternal, new-born and child health care at a South African university

**DOI:** 10.1186/s12909-020-02476-9

**Published:** 2021-01-07

**Authors:** Elizabeth Egieyeh, Mea van Huyssteen, Renier Coetzee, Angeni Bheekie

**Affiliations:** Discipline of Pharmacology and Clinical Pharmacy, School of Pharmacy, University of the Western Cape, Cape Town, 7535 South Africa

**Keywords:** Curriculum, Final year pharmacy students, Knowledge and skills, Maternal and child health care, Pharmacy education, South Africa

## Abstract

**Background:**

Maternal and child mortality is a global concern and one of South Africa’s quadruple burdens of disease. As easily accessible frontline healthcare workers, pharmacists play an important role in the continuum of maternal and child health (MCH) care according to recommendations by international health regulatory bodies. Pharmacy schools are obliged to train pharmacy students to meet the priority health needs of the population so that graduates are ‘fit for purpose’. The baseline study aimed to evaluate the knowledge and skills of 2017 final year pharmacy students who were exposed to a fragmented MCH care curriculum at a university in South Africa to inform curriculum review.

**Methods:**

A descriptive, quantitative, non-randomized study was conducted among final year pharmacy students using a self-administered structured questionnaire. The questionnaire was designed in sections to assess participants’ knowledge of reproductive and sexual health (RSH), maternal and antenatal care (MAC), neonatal and child care (NCC) and skills related to infant growth assessment procedures. Data was analysed descriptively using frequencies and percentages. A score of 50% in each section of the questionnaire indicated a pass. Participants assessed their exposure to MCH topics in the curriculum.

**Results:**

Of the 89 available students, 61% consented to participate in the study. The average scores attained for each section were; 62.4% for RSH, 54.5% for MAC, 50.4% for NCC and 25.3% for infant growth assessment. The pass rate was 78% for RSH, 56% for MAC, 57% for NCC, and 19% for infant growth assessment. About 13% of the participants passed all the knowledge and the skills sections. Age, gender, being a parent or doing locums did not have any influence on participants’ performance. Participants reported that they had more on-campus curriculum content exposure to RSH compared to other MCH care topics.

**Conclusion:**

Final year pharmacy students showed adequate knowledge of RSH with adequate curriculum exposure. Average knowledge of MAC, NCC and poor skills in infant growth assessment which corresponded to curriculum exposure was observed. The results suggest the need for improvement in the current curriculum in the affected areas to adequately equip students to render desirable services.

**Supplementary Information:**

The online version contains supplementary material available at 10.1186/s12909-020-02476-9.

## Background

Maternal and child health care is a continuum of integrated care which spans from adolescence or pre-pregnancy to delivery and childhood [1]. Maternal and child health care became a global concern with the high incidence of maternal and child mortality observed in the past three decades. Complications during pregnancy and childbirth are a leading cause of death. Globally, annual unintended pregnancies contribute to maternal and child mortality through 25 million unsafe abortions, 47,000 maternal deaths, 2.7 million neonatal deaths and 2.6 million stillbirths [2]. Most maternal and child mortality incidences are avoidable through provision of required care and management at every phase of the continuum of care [3].

The United Nations’ Sustainable Development Goals (SDG) 2030 has targets for reducing maternal mortality, under 5 mortality and providing universal access to sexual and reproductive health care services [3]. These targets are focal points that are aimed at increasing the global declines observed between 1990 and 2015 [3]. Sub-Saharan Africa and South Asia have the slowest reduction in maternal and child mortality rates and account for the highest burden of deaths in the world [3]. As part of sub-Saharan Africa, South Africa is one of the 74 countries globally that needs accelerated progress to meet the SDG targets [4]. In 2015, its maternal mortality rate declined to 121 per 100,000 live births while under-5 mortality rate stood at 30.2 but increased to 42 per 1000 live births in 2016 [5]. The observed decline was attributed to improved access to skilled health care professionals and healthcare services [3].

Causes of maternal and child mortality are classified either as direct or indirect obstetric causes. The World Health Organisation’s (WHO) International Classification of Diseases (ICD-10) for maternal mortality (ICD-MM) and perinatal mortality (ICD-PM) is intended to aid concentration on relevant interventions [6, 7]. Obstetric causes of neonatal mortality were preterm buwcirth complications, infections, intrapartum-related complications, congenital anomalies while post-neonatal mortality was attributed to pneumonia, diarrhoea, measles, and malaria [6]. Obstetric causes of maternal mortality included hypertension and infections in pregnancy, obstetric haemorrhage, septic abortions, and puerperal sepsis [7]. Pre-existing conditions which may be aggravated by pregnancy are classified as non-obstetric causes. These classifications have elevated the significance of obstetric and intrapartum causes of maternal and child mortality to the detriment of non-obstetric or indirect causes [8]. Consequently, the contributions of healthcare professionals who do not provide direct obstetric healthcare to mothers and children has been undermined and excluded from the recognized maternal and child health workforce [9].

Pharmacists as health care professionals are readily accessible to women and children at any point during the continuum of care, for preventative advice and prescription filling though they do not provide direct obstetric services and are not recognized as part of the maternal and child health workforce [9]. Their traditional role as medicine custodians also saddles them with the responsibility to ensure that critical medication and sterile surgical products or devices required for contraception, vaccines, pregnancy, labour and delivery are readily available at hospitals and clinics [10]. The roles of pharmacists in maternal and child health care is outlined in the International Pharmaceutical Federation’s statement of policy on the effective utilization of pharmacists in improving maternal, neonatal and child health across the continuum of care and in line with WHO suggested interventions to high-priority countries as it is currently experienced in practice [11]. Some of the services rendered which are also in line with the South African Pharmacy Council’s Good Pharmacy Practice [12] recommendations include reproductive health care (provision of and education on various contraceptive options including emergency postcoital contraception). Pre-pregnancy care (folic acid) for women and adolescents [13], advising on the implications of pre-existing health conditions on pregnancy [14, 15], pregnancy tests, ensuring medication safety and managing simple pregnancy related conditions [13, 16] nutritional advice in pregnancy, and malaria prevention [17] are some of the other services. Baby and child health care (encouraging exclusive breastfeeding, immunization and adequate nutrition for infants and children) [18], and identifying women at risk of post-partum depression [15]. Health promotion and prevention (i.e. promoting a healthy planned pregnancy and preventing unintended pregnancies) is thus the core of pharmacists’ service provision in maternal and child health care.

The South African Pharmacy Council regulates pharmacy education and practice in South Africa. The high rate of maternal and child mortality in the country warrants that all healthcare professionals who participate in maternal and child health care delivery are adequately trained to render quality and effective services that meet the needs of these group of patients. The South African Pharmacy Council’s Good Pharmacy Practice manual stipulates the minimum professional standards required to provide maternal and child health care services in a pharmacy including standards for the physical facility and equipment [12]. Pharmacists are expected to know the procedures, interpretation, provision and explanation of results for each test carried out. Documentation, record keeping and confidentiality are stated as essentially parts of service provision. The need for adequate training that confers required knowledge and skills as crucial for the provision of these services is highlighted. Registration of postgraduate training with the South African Pharmacy Council is mandated for the provision of reproductive health care services. A reason for the current Good Pharmacy Practice standards is in preparation for the pharmacist’s role in the National Health Insurance (NHI) [12]. The NHI is the official South African policy to attain universal health coverage for all South Africans. It is posited on primary health care reengineering, which involves a change of focus from curative to health promotion and prevention [19], a role that pharmacists are trained to embrace.

Although pharmacists are in a position to positively influence pharmacotherapy and consequently the wellbeing of mothers and children, they often feel ill equipped to render such services. In an online cross-sectional, cross-country survey comparing the knowledge, perception and training opportunities of practicing and resident pharmacists in Canada, Qatar and Uganda, the overall knowledge in maternal and child health care was noted to be low at 53.7% [20]. A Nigerian study reported that although a high client load of mothers and children was established at community pharmacies, community pharmacists’ baseline knowledge was inadequate to provide required services [21]. A Lithuanian study of 27 community pharmacists reported that they lacked adequate experience to advise pregnant women but they were more effective if the patient was an acquaintance [16]. Most community pharmacists alluded to the fact that their entry-level degree did not prepare them adequately for this specialty service [20]. In addition, lack of continuing professional development in maternal and child health care was mentioned as a hindrance to knowledge enhancement [20]. Indeed, access to resources for information varied among participants, resulting in different information being proffered for the same conditions [18]. Lack of knowledge about the safety and use of certain medications and absence of clinical data for herbal remedies during pregnancy hindered pharmacists from providing required counselling to patients [16].

In a study conducted at a Pakistan medical school, the reduction of high maternal and child mortality rate was hypothesized to be linked to undergraduate medical students maternal and child health care curriculum content exposure. Non-contextualized, inadequate and fragmented teaching methods were thought to be responsible for the knowledge and skills gap observed in medical graduates [22]. Similarly, in Zimbabwe, the neonatal mortality rate persisted at 29 per 1000 live births (1999 and 2015) despite a decrease (102 to 75) in under-five mortality rate in the same period. Further, an assessment of the state of healthcare facilities and quality of available service indicated significant knowledge and skills gaps among health workers in managing common new-born conditions [23].

Although several studies have evaluated the knowledge and skills of practicing pharmacists in maternal and child health care, no documented study has investigated undergraduate pharmacy students’ knowledge and skills in this area or the adequacy of the undergraduate curriculum content to provide such knowledge and skills. This paper explores how undergraduate pharmacy education at a higher education institution in South Africa is preparing students to meet the maternal and child health care needs of the nation, one of the country’s quadruple burdens of disease. The baseline study aimed to evaluate the knowledge and skills of 2017 final year pharmacy students who were exposed to a fragmented maternal and child health care curriculum at a university in South Africa to inform curriculum review.

## Methods

### Study design

The descriptive, quantitative, non-randomized study assessed final-year undergraduate pharmacy students’ knowledge and skills in maternal and child health care, and their self-reported views on curriculum content exposure.

### Setting

The University of the Western Cape hosts the only School of Pharmacy in the Western Cape province of South Africa. The School has four main academic disciplines, namely pharmaceutics, pharmacy practice, pharmaceutical chemistry and a combination of pharmacology and clinical pharmacy along with an experiential learning component. The curriculum is presented in modules per semester for each discipline. As such, a module topic would be covered asynchronously or non-systematically in the different disciplines over the four-year study period, thereby fragmenting student learning. In addition to the on campus theoretical and laboratory-based teaching, the school’s experiential learning programme, Service Learning in Pharmacy (SLiP), aims to provide real world experiences for students to apply their theoretical knowledge [24]. An externship experience, also allows students to clock hours in pharmacies during their vacation periods. Also, some students opt to work as pharmacist’s assistants after their second year of study to earn an income or gain experience, which is not a school requirement.

In 2013, the South African Pharmacy Council called for a curriculum change across pharmacy schools to redesign the curriculum towards clinical competencies required for patient centred pharmaceutical care [25]. In the pre-2013 curriculum, components of maternal and child health care such as contraception (with a contraception demonstration practical), pregnancy care, infant care, communicable diseases and immunization lectures were embedded in the discipline of pharmacy practice in the third year of study. The post-2013 curriculum required all clinical components previously taught in the pharmacy practice discipline to move to the new discipline of pharmacology and clinical pharmacy (known as the discipline of pharmacology before 2013). Subsequently, the theoretical teaching of maternal and child health care moved between disciplines, from pharmacy practice (module PHA324) to pharmacology and clinical pharmacy (module PHC223). However, the reduced class time to cover all the maternal and child health care topics led to contraception remaining as a formal lecture while the rest of the topics were taught as a 2-h crash course (Table 1). Any other exposure to these topics may have been references to the importance of health education and medication safety in pregnancy and paediatrics during pharmacotherapy lectures. Maternal and child health care curriculum content exposure was concluded with more contraception lectures in the first semester of the 4th year of study.

**Table 1:** Maternal and child health curriculum content by year of study. Pre-2013 and 2013–2016 at the School of Pharmacy

Year of study	Pre-2013		2013–2016		
	3rd year	4th year	1st year	2nd year	4th year
**MCH theoretical content**	Contraception part 1 Pregnancy care, infant care, communicable diseases, immunization	Contraception part 2	Environmental and nutritional health (diarrhoeal disease, de-worming, Vitamin A supplementation, pregnancy supplements	Contraception part 1 Pregnancy and infant care, communicable diseases, immunization (2 h crash course)	Contraception part 2
**Module code /discipline**	PHA324/ Pharmacy practice	PHA414/ Pharmacy practice	PHC123/ Pharmacology and clinical pharmacy	PHC223/ Pharmacology and clinical pharmacy	PPR 414/ Pharmacy practice
**SLiP activity** **/duration**			ORS practical (in collaboration with Pharmaceutics discipline) /1 h Community visit/3 h	PHC Facility MCH-SLiP/ 9 h	

Key: *SLiP* Service learning in pharmacy, *SLiP-MCH* Service learning in pharmacy-maternal and child health

The School took the new curriculum as an opportunity to embed relevance in its service learning programme and as such expanded it across all 4 years of study (previously only presented in the third and fourth years of study) [24]. SLiP was attached to pharmacology and clinical pharmacy modules in first, second and fourth year, and a pharmacy practice module in the third year, as it was intended to cut across disciplines to offer integrated, real world learning to the students. A dedicated service learning programme on maternal and child health care was developed for second year students in the second semester (attached to PHC223). This programme required student to perform maternal and child health care activities in public primary health care facilities under the supervision of facility nurses. Prior to this, the environmental health service learning programme in the second semester of the first year (attached to PHC123) focussed on diarrhoeal disease, one of the communicable diseases associated with neonatal and child mortality. This programme required students to prepare dry mixtures of oral rehydration salt and sugar prepacks under the supervision of South Africa Pharmacy Council registered faculty [25]. The dry formulation sachets prepared by the first-year students were distributed to the primary health care facilities by the second-year students who undertook their SLiP maternal and child health care programme in the same semester. At the facilities, second year students were expected to carry out health education activities that promote the health and wellness of mothers and infants, educate mothers on medicine administration (nevirapine, and vitamin A drops, oral rehydration solution (ORS) and deworming agents), participate in infant growth assessment, chart and interpret information in the infant road to health booklet under the direct guidance of a facility nurse.

### Study participants

The target audience for the study were final year pharmacy students registered at the School of Pharmacy, University of the Western Cape in 2017. Out of the 102 registered students, five were recruited as student researchers leaving 97 students as possible study participants. The inclusion criterion was registration as a full final year student. Participants were excluded if they had outstanding modules to complete in either their third or second year of study.

### Questionnaire development

A paper based structured questionnaire (see supplementary file for the questionnaire) which comprised of four main sections A, B, C and D was developed for the study thorough literature review of related published studies [11, 18, 20, 22]. Section A covered participants’ demographic details which included age, gender, locum experience (a person who substitutes for another person from the same profession to temporarily fulfil their duties) and parenting status (if participants were parents). Section B (28 items) comprised of the knowledge section with three sub-sections: reproductive and sexual health which focused on contraception (9 items, 9 marks); maternal and antenatal care which covered preconception and pregnancy care (10 items, 17 marks), and lastly, neonatal and child care, which included infant care and nutrition, childhood diseases and immunization (9 items and 12 marks). Section C (6 items, 8 marks) consisted of the skills section which assessed students’ understanding of infant growth assessment procedures and its relevance. Sections B and C generated the scores for evaluation of participants’ knowledge and skills with 34 items (46 marks) made up of multiple-choice (one mark per question) and short answer questions (one mark per response). Multiple-choice questions had the option of ‘I don’t know’ to minimize guessing [20]. Section D assessed participants’ maternal and child health care curriculum content exposure (curriculum assessment tool) over their undergraduate study period in order to contextualise their knowledge and skills evaluation outcomes.

### Pilot study

A convenient sample of eight students participated in the pilot study. The pilot questionnaire was administered in the form of a closed book written test and which took place in a predetermined campus venue supervised by invigilators. Participants were required to answer all the questions and also assess the items in the questionnaire for validity, reliability, clarity and conciseness on a post-study questionnaire after the study questionnaire was completed and handed in to the invigilators. Questionnaire completion required about 45 min.

Analysis of the pilot study and post-study questionnaire revealed that of the eight students who participated in the pilot test, some found the questions clear and unambiguous (*n* = 5) while the others found some questions to be vague (*n* = 3). Participants’ opinion was equally divided on whether there were too many questions or not. Most of the participants found the questions difficult and struggled to understand some abbreviations such as MUAC (mid-upper arm circumference) and terms like mastitis (*n* = 5). Generally, the participants agreed that the questions were basic; however, they struggled to answer some questions because they were not exposed or adequately exposed to certain maternal and child health care topics or they had forgotten some of the content. Participants suggested that the number of questions should be reduced, more immunization questions should be included and the skills assessment section should be omitted as in their opinion, it was not relevant to a pharmacist’ scope of practice. They suggested that the instructions on the curriculum assessment tool (Section D) should be simplified. All the participants stated that the time given was sufficient to read the consent letter, complete the questionnaire and curriculum assessment tool. All of the participants found the information sheet and consent form easy to read and understand. The majority of the participants found a 50% pass mark in each knowledge subsection and skills section to be reasonable. A 50% score is the minimum pass mark for most modules at the university and it represents average lowest permissible knowledge of module content. A final questionnaire was prepared based on the comments from the pilot test participants, and additional comments and suggestions of four faculty members.

#### Validity and reliability

The content validity of the 34-item questionnaire was reviewed and verified by four faculty members who were knowledgeable in the subject area, five fourth year students recruited as researchers for this study and eight students who participated in the pilot study. They assessed the spread of the items, appropriateness, readability and clarity. However, a pilot study was carried out with one group of subjects to measure the questionnaire once and test its reliability. Although scoring agreement was not calculated but it was ensured by training the scorers on what do when grading the instruments, providing a moderated memorandum and ensuring that graded questionnaires were moderated. Unresolved discrepancies among scorers was fixed by the main author who authenticated all the graded questionnaires. In addition, to ensure reliability, questionnaire administration was carried out during a regular lecture time slot, timed and supervised appropriately. The content covered in the questionnaire was part of a university module and it served as a tool to assess the knowledge and skills acquired by participants from lectures. The medium of communication was English which is also the official language used at the university.

### Recruitment and data collection

The final year students were informed of the research through verbal announcements during class periods and e-mails sent through the university’s electronic communication platform. They were encouraged to participate in the study as the outcome would help to improve student learning in maternal and child health care at the School. Students were informed that their non-participation in the study would have no negative consequences towards their course assessment, as participation was completely voluntary. During a routine lecture period requiring the attendance of all final-year students at a designated venue, 54 of the available students excluding those who participated in the pilot study consented to participate in the study and they were provided with study information sheets and consent forms. Thereafter, the questionnaire was handed out and self-administered. Participants were asked not to use the internet or consult with each other during the study which was timed for 60 min and supervised.

### Data analysis

Three student researchers marked the completed questionnaires using a structured memorandum; thereafter two student researchers moderated the marked questionnaires before the marks were finalised and captured. Data was captured on an Excel spreadsheet and exported into the Statistical Package for the Social Sciences (SPSS) version 26 for analysis [26]. Data was analysed using participants’ scores in the knowledge and skills sections descriptively as frequencies (n), percentages (%) and mean scores. Spearman’s correlation (r) was conducted to explore the strength of the relationship between ranked continuous variables. Chi- square was used to compare the relationship between categorical variables (demographic characteristics and students pass (≥50%) and fail (< 50%) categories in reproductive and sexual health, maternal and antenatal care, and neonatal and child care). Student exposure to maternal and child health care curriculum content was determined descriptively in frequencies and percentages.

## Results

On excluding the eight students who participated in the pilot study, 89 registered final year students were available for the main study. About 61% of them participated in the study. Two-thirds of the participants were female (63%) and 98% of participants’ age ranged between 20 to 30 years. Eight (15%) participants were parents while over half (59%) did locums as post basic pharmacist’s assistants. Six of those who did locum duties had more than 2 years’ experience. Table 2 shows the demographic characteristics of participants.

**Table 2:** Demographic characteristics of participants (*N*=54)

Demographic data	Number of participants ***N***=54 (%)
**Gender**	
Female	34 (63)
Male	20 (37)
**Age (in years)**	
20 to 30	53 (98)
31 to 40	1 (2)
**Parenting status**	
Have children	8 (15)
No children	46 (85)
**Locum**	
Yes	32 (59)
No	22 (41)
**Locum experience (in years)**	
1 to 2	26 (48)
> 2	6 (11)

Table 3 summarises the descriptive statistics for the participants’ scores for each section of the questionnaire. Kolmogorov-Smirnov test of normality showed that the distribution of scores for the knowledge and skills sections was evenly split between normal (*p* =.025, *p =.065*) and non-normal distribution *(p* =.000) leading to the use of non-parametric tests for data analysis to avoid transforming variables for parametric tests. The mean scores were 62.4% in reproductive and sexual health, 54.5% in maternal and antenatal care, 50.4% in neonatal and child care and 25.3% in written assessment of participants’ skills and understanding of the relevance of infant growth assessment.

**Table 3:** Descriptive analysis of participants scores in percentages (*N*=54)

	Reproductive and sexual health	Maternal and antenatal Care	Neonatal and child Care	Skills
**Mean**	62.4	54.5	50.4	25.3
**Median**	67	55	50	16
**Mode**	67	36	33	13
**Std. Deviation**	16	17.8	20.5	20
**Minimum**	22	27	0	0
**Maximum**	89	91	92	88
**Percentiles 25**	55	36	33	13
**75**	78	73	67	28.3
***P*-value**	.000	.025	.062	.000

*P*-value=Kolmogorov-Smirnov test of normality.

Table 4 shows the number of participants who scored above or equal to 50% in each section of the questionnaire. The pass rate for the knowledge section was above 50% [reproductive and sexual health (78%), neonatal and child care (57%), maternal and antenatal care (56%)] while the skills section recorded a pass rate of 19%. An overall average score of more than 50% was achieved by 44% of the participants. Excluding the scores for the skills section form the overall average score resulted in 65% pass rate. About 13% of participants scored above 50% in every section.

**Table 4:** Number of participants who scored ≥ 50%

	Number of participants that scored ≥ 50% ***N***=54 (%)
Reproductive and sexual health	42 (78)
Maternal and antenatal care	30 (56)
Neonatal and child care	31 (57)
Skills	10 (19)
In each knowledge subsection and skills section	7 (13)
Overall average score	24 (44)
Overall average score excluding skills section	35 (65)

Table 5 indicates the number of participants who had the right responses per question. More participants gave the right responses to reproductive and sexual health questions (61%) while infant growth assessment skills recorded the least number of participants with the right responses (17%). Both maternal and antenatal care, and neonatal and child care had an equal number of participants with the right responses (44%).

**Table 5:** Number of participants with the right response per question

	N (54)	%	Median of the item
**Reproductive and sexual health**			61
1. Which hormones are present in combined oral contraceptives?	54	100	
2. When should combined oral contraceptives be started?	11	20	
3. Which contraceptive methods’ effectiveness relies on the client’s ability to use them correctly?	53	98	
4. Which contraceptives are Long acting reversible contraceptives (LARC)?	28	52	
5. When may the use of emergency contraception (EC) be indicated after sexual intercourse?	21	39	
6. Which information should the pharmacist obtain from the patient before emergency contraception is dispensed?	33	61	
7. Why is dual contraception encouraged?	10	19	
8. Which one of the following is true about oral progestogen-only pills?	51	94	
9. Rifampicin, Lopinavir/Ritonavir, Nevirapine are enzyme inducers that interact with oral contraceptives to do cause which effect?	40	74	
**Maternal/antenatal care**			44
1. All non-pregnant women of reproductive age should be advised to commence periconceptual folic acid supplementation (women planning pregnancy)	38	70	
2. What is the least number of antenatal clinic visits that every pregnant woman should attend?	18	33	
3. State 4 lifestyle modifications recommended for pregnant woman for a healthy pregnancy and baby?	48	89	
4. List 4 signs that a pregnancy is in danger	51	94	
5. When is antiretroviral (ARV) therapy initiated in newly diagnosed HIV positive pregnant women?	46	85	
6. Identify the non-teratogenic medicines on the list	8	15	
7. Which factors influence the manifestation and severity of teratogenicity?	27	50	
Explain the cause, pharmacological and non-pharmacological treatment of the following common conditions of pregnancy			
8. Morning sickness	24	44	
9. Heartburn^a^	11	20	
10. Vaginal thrush	24	44	
**Neonatal and child care**			44
1. Exclusive breastfeeding (EBF) is defined as giving only breast milk to infants for the first -------------------- months of life.	43	80	
2. An HIV exposed infant is one whose mother is HIV infected or whose HIV infection has not been confirmed or excluded. Which ARV medication is given to such infants at birth?	42	78	
3. WHO recommends that HIV positive women who are on ART should exclusively breastfeed their babies. True or false?	36	67	
4. What causes cracked nipples during breastfeeding?	9	17	
5. The Expanded Programme on Immunization (EPI) covers the major killer diseases of infanthood. List three of such diseases^b^.	14	26	
6. How is diarrhoea treated in infants and children?	44	81	
7. Deworming agents are initially given to children at what age and at what interval subsequently?	24	44	
8. Outline one pharmacological and non-pharmacological treatment for diaper/nappy rash^a^	6	11	
9. How is mastitis treated?	11	20	
**Skills assessment (Infant growth assessment)**			17
1. Explain how an infant’s (0–12 months) height is measured^a^?	4	7	
2. Should an infant be fully clothed or undressed during weight measurement?	49	91	
3. How is an infant’s head circumference measured^a^?	1	2	
4. Why is an infant’s head circumference measured?	17	31	
5. What is MUAC?	9	17	
6. Why is MUAC measured?	15	28	

*MUAC* Mid upper arm circumference. *HIV* Human immunodeficiency virus

^a^ indicates the question counted for 2 marks and only participants who scored 2 marks are represented on the table

^b^ indicates the question counted for three marks and only participants who scored 2 marks are represented on the table

**Fig. 1:**
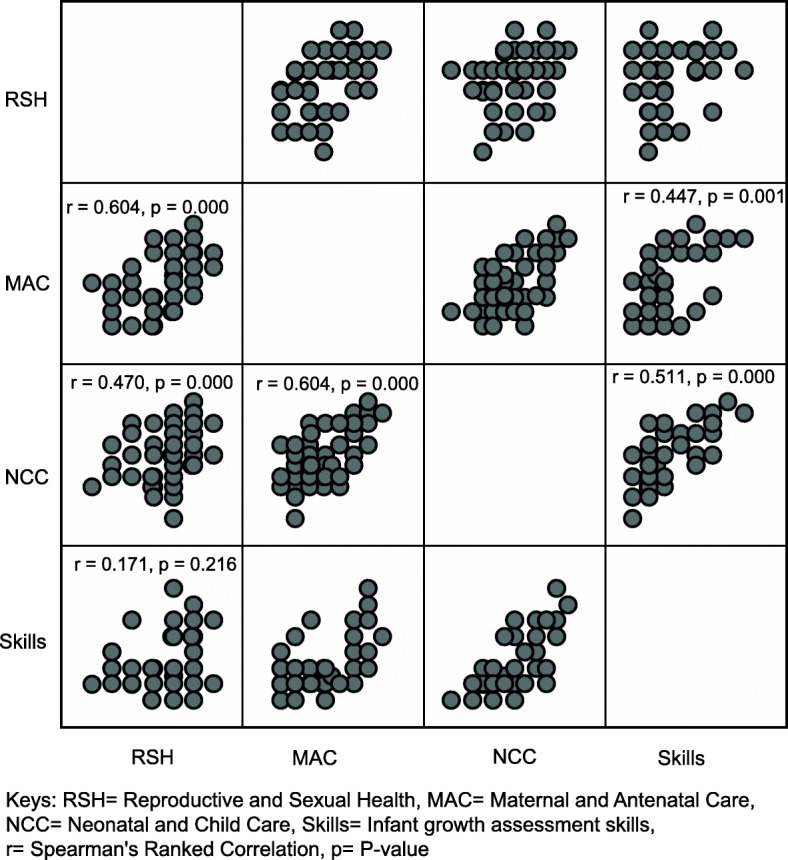
Scatterplot matrix showing correlation among participants’ ranked knowledge and skills mean scores. Statistically significant correlations were observed between maternal and antenatal care, and neonatal and child care (*r*=0.604, *p* =.000) reproductive and sexual health, and maternal and antenatal care (*r*=0.604, *p* =.000), neonatal and child care, and infant growth assessment skills (*r*=0.511 *p* =.000)

In Fig. 1, a strong positive correlation (a corresponding increase) was observed in participants’ knowledge of maternal and antenatal care, and neonatal and child care (r = 0.604); maternal and antenatal care, and reproductive and sexual health (r =0.604), neonatal and child care, and infant growth assessment skills (r = 0.511). Doing a locum, being a parent, gender or age had no significant effect on participants’ knowledge and infant growth assessment skills (*p* > 0.05).

The result of participants self-reported curriculum content exposure is shown on Table 6. About 81% (*n* = 44) of the study participant completed the assessment tool. More participants (95%) reported on-campus exposure to reproductive and sexual health topics compared to maternal and antenatal (55%) and neonatal and child care (57%). During SLiP at the primary health care facilities, less participation occurred in neonatal and child care (67%) compared to infant growth assessment activities (71%). Most participation (93%) was observed in maternal and antenatal where promotion of mother and infant health and well-being was carried out.

**Table 6:** Self-reported curriculum content exposure assessment (*N*=44)

Topic	Focus	N (44)	%	Median of the item
**Reproductive and sexual health**				95
	Contraceptive methods available in South Africa	44	100	
	How the methods work	43	98	
	Efficacy, when effective, duration of use	41	93	
	Advantages and disadvantages	42	95	
	Potential short term and long-term effects	40	91	
	Special precautions	41	93	
	Possible problems to report	41	93	
	Return to fertility	43	98	
	Post coital contraception	42	95	
**Maternal and antenatal care**				55
	Folic acid & Fe Supplement for pre-pregnancy	28	64	
	HIV therapy provision pre-pregnant	31	70	
	Lifestyle modifications for pregnant women	25	57	
	Deworming agents during pregnancy	13	30	
	Tetanus immunization protocols during pregnancy	13	30	
	Treatment of infection (UTI, STI, etc.)	32	73	
	Importance of antenatal visits	21	48	
	HIV treatment during pregnancy	39	89	
	Choices for unwanted pregnancy (abortion, adoption)	22	50	
	Maternal danger signs in pregnancy	22	50	
	Medicines contraindicated in pregnancy	30	68	
	Common conditions in pregnancy, management/treatment	19	43	
	Role of pharmacists in pregnancy care	24	55	
	Postpartum depression	10	23	
	Suitable contraceptive methods	34	77	
	Medicines contraindicated in lactating mothers	30	68	
	Management of common breastfeeding conditions	18	41	
**Neonatal and child care**				57
	Exclusive & complementary breastfeeding	39	89	
	Breast milk substitutes complementary feeding options	19	43	
	Children immunisation protocols	25	57	
	Infant and children vitamin supplementation	25	57	
	Treatment of infections in children (HIV, pneumonia, etc.)	29	66	
	Treatment of diarrhoea in children	37	84	
	Improving of drinking water	28	64	
	Deworming	20	45	
	Medicines contraindicated in infants	20	45	
	Common infant problems, management/treatment	15	34	
	Role of pharmacists in infant care	18	41	
**Service learning in pharmacy (SLIP)**				
Maternal and antenatal care	Exclusive breastfeeding	38	86	93
	Lifestyle and nutrition	36	82	
	Safe sex practice	41	93	
	Contraceptive use	43	98	
	Handwashing, personal hygiene	41	93	
Neonatal and child care	Nevirapine administration to infants	31	70	64
	Oral rehydrate solution	40	91	
	Vitamin A supplementation	23	52	
	De-worming agent	21	48	
	Immunisation protocol	28	64	
	Assess infant’s dehydration status	31	70	
	Advice when infant vomits therapy	17	39	
Infant growth assessment	Infant weight, height taken	33	75	71
	Interpret/ evaluate growth	29	66	

*UTI* Urinary tract infection. *STI* Sexually transmitted infection

## Discussion

The study assessed the knowledge and skills of final year pharmacy students in maternal and antenatal care against the backdrop of the high mortality rate recorded in sub-Saharan Africa and its occurrence as one of South Africa’s quadruple burdens of disease. The country is optimising its health workers roles in maternal and child health care in order to achieve the targets of SDG 3 for 2030 through the reengineering of the primary health care system. Pharmacists as easily accessible health care providers especially in the community where 71% of graduates are employed and are an overlooked and underutilised resource in the fight to reduce maternal and child mortality rate [27]. Nevertheless, like with other health care professionals, adequate undergraduate and post qualification training are necessary to ensure competency in service provision. A few studies have assessed pharmacist’s knowledge in maternal and child health care or its individual components. To our knowledge, no published study has assessed pharmacy students’ knowledge and skills in maternal and child health although some work has been done on its individual components: reproductive and sexual health, maternal and antenatal care, and neonatal and child care [28–33].

Despite adequate notifications and information, about 61% of final year students consented to participate in the study. This may have been due to their busy schedule as final year students, or the fact that participation in the study did not contribute toward their academic grades, or apathy toward the research topic as not being very relevant to pharmacists. These opinions are shared in a guide for recruiting higher education students for research published by the Higher Education Quality Council of Ontario where student participation in research was indicated to be based on altruism, project related interest or self-centredness [34]. The self-centeredness of student participation may have been expressed in this study as more females (63%) aged between 20 to 30 years were interested in participating in the study probably in preparation for motherhood compared to males of the same age range. Alternatively, the higher female participation could be in line with the class gender distribution as observed in a demographic study of the final year class of 2015 [35]. Similarly, more female respondents were recorded in some studies of community pharmacist knowledge and service provision in maternal and child health care in the United States of America (USA), and a multi-country survey of Canada, Qatar and Uganda [20, 35]. The skewed interest in research participation does not indicate that females have better knowledge and skills in maternal and child health care as the current study found no association among knowledge scores and age, gender, parental or locum status. Similarly, a multi-country study of pharmacists offering maternal and child health care services showed that age, gender, parental status had no significant effect on participants’ knowledge and skills. However, pregnant or lactating women were more comfortable to discuss their concerns with female than male pharmacists according to a Kuwait study of community pharmacists’ rendering services to pregnancy and breastfeeding women [18].

### Participants’ knowledge of reproductive and sexual health

The reproductive and sexual health knowledge tested in this study focused mainly on contraceptive methods. The relevance of which is reflected in South African pharmacy practice as pharmacists are allowed to initiate and offer comprehensive reproductive health service after obtaining the necessary training and registering such supplementary training with the South African Pharmacy Council [12]. However, patients do not ask if pharmacists have supplementary training when consulting them for general reproductive and sexual health advice or information. As a result, undergraduate training should provide fundamental knowledge and skills in this area. Similarly, more than ten states in the USA have endorsed legislation permitting pharmacists to directly provide contraceptives to patients and studies assessing curriculum content and student knowledge are available [28, 29]. The impact of contraception on maternal and child mortality can be emphasized by a 2019 estimate from the Family Planning 2020 report for South Africa which estimated that if about 7.9 million women are on modern contraceptives, 2.9 million unintended pregnancies, 265,000 unsafe abortions and 2500 maternal deaths can be averted [36].

Assessment of the contraception knowledge and skills of participants in our study showed a mean score of 62.4% (median 67%), whereby over three-quarters.

(78%, *n*=42) of the participants had scored 50% and above. However, the high standard deviation (SD) of 16 shows that scores were widely spread out from the mean with an interquartile range (IQR) of 55–78%. Although the variation in reproductive and sexual health knowledge was wide amongst participants, 61% of the participants gave the right responses to the questions, and as such they may be relied on to render moderately adequate services to patients. This can be attributed to the comprehensive curriculum exposure as reported by 95% of the participants in the second and fourth year of study which included contraceptive products application demonstration session. Similarly, a pre-post intervention study carried out by El-ibiary et al. demonstrated that using multiple teaching methods for reproductive and sexual health improved students’ knowledge and confidence [28]. In addition, some studies have shown that a dispersed teaching approach, like the one used in this curriculum, by spreading the content for contraceptives between second and fourth year, enhanced long-term knowledge retention [37, 38].

### Participants’ knowledge of maternal and antenatal care

Maternal and antenatal care included preconception care in this study. The South African Maternal Care Guidelines (2015) encourages all health workers who care for women of reproductive age to offer preconception care [39]. The guideline also explains that antenatal care aims to ensure that women have a safe pregnancy and healthy babies. Pharmacists and pharmacy students need to have adequate knowledge and skills as they often offer care and advice to women of reproductive age and pregnant women at different health care settings. Participants’ mean and median scores for maternal and antenatal care was 54.5%, slightly above the minimum allowed pass mark of 50% with 56% (*n* = 30) of the participants scoring 50% and above. Participants’ scores were widely dispersed from the mean (SD 17.8) and showed a slightly above average knowledge of maternal and antenatal care content which was less than reproductive and sexual health content. The unexceptional knowledge observed for study participants may be attributed to the 2-h single exposure to relevant curriculum content in the second year of study which was brief, and occurred 2 years prior to the study commencement. The effect of teaching methods on long term knowledge retention may have resulted in about 47% of the participant being able to give the right answers to the questions in our study. This was further highlighted in a study of 133 third year pharmacy students enrolled in a 6 years Doctor of Pharmacy programme at a private, midwestern university [30]. Participants’ knowledge of folic acid and neural tube defects based on the online education was assessed using pre-post intervention longitudinal survey. At baseline, 50% of the participants could correctly answer 5 out of the 10 questions. The intervention was a 30 min video followed immediately with a post-test which showed statistically significant improvements in the response of the participants to nine of the 10 questions. However, a longitudinal test carried out 9 months after the post-test indicated a drop-in participants’ performance relative to the post-test but an increase compared to baseline knowledge with statistically significant increases in six of the nine questions relative to baseline. The result demonstrated the inadequacy of the online, one-time exposure for long term knowledge retention [30]. Curriculum content exposure assessment in the present study showed that about 55% of participants acknowledged receiving lectures on maternal and antenatal care. However, this contradicts the SLiP experience where 93% participation was recorded in health and wellness promotion for maternal and antenatal care. The interaction could have enhanced participants’ performance as alluded to by Bheekie et al. in their study of the opportunities for student learning and service delivery in SLiP where they explained that it enabled contextualized learning [24]. A study evaluating the University of Arizona College of Pharmacy’s curriculum and the knowledge and abilities of pharmacy students to counsel pregnant and breastfeeding women about the use of over-the-counter products and prescription medications revealed that the curriculum was deficient in some areas such as teratogenicity and comprehensive case studies. The lack of knowledge and abilities was reinforced by Arizona College of Pharmacy study participants’ indication of their inadequacy to render services to this patient population regardless of their year of study [31].

### Participants’ knowledge of neonatal and child care

The ability of pharmacists to recognise the symptoms of common childhood illnesses like diarrhoea and the importance of early referral would contribute immensely to the reduction of infant and child mortality. Knowledge of relevant, age specific nutrition and immunization protocol would be invaluable to service provision. Participants in the current study had a mean score of 50.4% in neonatal and child care. About 57% (*n* = 31) scored above 50% with wider dispersion from the mean (SD 20.6) compared to reproductive and sexual health, and maternal and antenatal care. Neonatal and child care had the highest maximum score of 92% but a minimum score of 0% which supports the high score dispersion observed (IQR 33–67%). Participants’ close performance in neonatal and child care (50.4%) and maternal and antenatal care (54.5%) may be attributed to the two topics being taught during the 2-h single exposure in the second year of study. This is further supported with the observation that almost the same number of participants gave the right answers to neonatal and child care and maternal and antenatal care questions respectively (44, 47%). In addition, the similarity in participants’ performance was also statistically supported as significant because an increase in participants knowledge of neonatal and child care was shown to be positively correlated to same in maternal and antenatal care (r = 0.604, *p* < 0.005). However, the slight difference between the mean score for neonatal and child care (50.4%) and maternal and antenatal care (54.5%) may be attributed to higher student participation in maternal and antenatal care (93%) observed during SLiP which may have helped to contextualize student learning and aid knowledge retention. Similarly, a study involving final year students in all pharmacy programmes in Jordan showed that 62.1% of the 354 participants scored two or less out of five in the realistic case scenarios used to assess knowledge of paediatric treatment and dosing with 81.1% of the participants indicating that more paediatric related topics should be included in the curriculum. About 44% of participants self-reported competency in paediatrics for future practice. Absence of adequate hands on practical training was also insinuated to have contributed to the deficiency in participants knowledge [32]. The importance of paediatric training in USA Colleges and Schools of Pharmacy is emphasized by the recommendation made by the Paediatric Practice and Research Network (PRN) of the American College of Clinical Pharmacy in 2005. PRN’s recommendation included a minimum of 25 h of classroom instruction in paediatrics from the first year of the PharmD programme, an elective course consisting of 16 to 32 contact hours, and at least 1 practice experience in paediatrics. Pharmacists in the USA can be certified as immunizers in addition to providing information about immunizations as one of their expanded roles [33]. Similarly, South African pharmacists can be certified as immunizers with the appropriate training but the establishment of baby wellness clinics run by nurses in most retail chain pharmacies discourages most pharmacists from acquiring this skill [12, 40].

Infant and young child feeding is a major part of neonatal and child care and exclusive breastfeeding (EBF) has been shown to have global advantages to mothers, babies and the community. The global risk of death to exclusively breastfed infants has been estimated to be 12% less compared to that of non-breastfed infants and 13.8% of deaths of children below 2 years can be averted by EBF [41]. Universally, EBF has been shown to prevent 20,000 breast cancer deaths in mothers annually [42]. Sadly, about 32% of South African babies were exclusively breastfed in 2016 despite the 2012 policy which expects all health workers to promote EBF for 6 months irrespective of the human immunodeficiency virus (HIV) status of the mother. Interestingly, about 80% of participants in our study understood how long a baby should be exclusively breastfed. However, Edwards reported in his study that there were a few available studies on pharmacists’ education and knowledge regarding breastfeeding. While most of them focused on medication and lactation, they lacked information on infant feeding guidelines [43]. To address the gap, he created and piloted an online breastfeeding tutorial for pharmacy students at three schools of pharmacy in the USA. A survey of students’ attitudes regarding their roles in infant feeding on completion of the online tutorial revealed that 78% of participants recognised the importance of their role in providing advice about meeting the nutritional needs of infants and supporting parent’s infant feeding choices (86%). A few participants lacked or had little confidence that they could answer questions from parents on breastfeeding their infants (14%) and the introduction of solid foods to their infants (24%).

### Participants’ infant growth assessment skills’ knowledge

Participants in our study showed poor ability to assess infant growth with a mean score of 25.3%, well below the minimum permissible pass mark of 50% with 19% (*n* = 10) scoring 50% and above. About 17% of the participants gave the right answers to the questions in this section. Participants’ poor knowledge of infant growth assessment processes and its relevance may have been affected by the lack of on-campus practical exposure. However, despite the lack of on-campus practical exposure, 71% of the participants self-reported carrying out this assessment at the primary health care facilities during the second year SLiP programme which should have imparted some knowledge and skills to them at the time. This assumption is supported by the correlation observed between participants knowledge of neonatal and child care and infant growth assessment skills in our study result (r = 0.511 *p* < 0.005), that an increase in one would lead to an increase in the other. A plausible reason for the large difference between participants mean scores may be assumed to be as a result of inadequate curriculum exposure and knowledge decay over time. A prospective study of fourth year medical students’ knowledge retention with regard to communication with patients and the physical examination after an introductory course in basic paediatric cardiology at a university in Brazil showed knowledge decay over time. Students’ knowledge of the basis of clinical skills, diseases and pathophysiology mechanisms was found to decrease progressively from the initial score obtained immediately after the lecture, 6 months and a year later. Knowledge decay was said to occur due to lack of use [44].

Participants in our pilot test had advised that the skills section be excluded from the questionnaire as it was not relevant to pharmacists. However, since 2016, the South African Pharmacy Council recommended that second year pharmacy students should have hands-on training at primary health care facilities during the SLiP maternal and child health care programme to enhance skill development in child care. The purpose of infant growth assessment skills is to determine whether a child has a growth problem (i.e. over or underweight), which sometimes points to a nutrition-based origin that should be addressed as early as possible to prevent lasting health complications [45]. The ability of pharmacists to provide practical and measurement-based proof validates the information provided to the patient. In addition, the South African Pharmacy Council’s Good Pharmacy Practice requires pharmacists providing baby and child health care to be informed about childhood problems and the importance of early referral [12].

Overall, about seven (13%) out of the 54 participants passed all the sections of the assessment by scoring 50% and above in the knowledge and skills sections. This is a poor reflection of participants’ knowledge and skills in maternal and child health care. When scores from the different sections were added together, 24 (44%) of participants had an average score of 50%. Although the ‘pass mark’ was set at 50% in this study, a study that compared pharmacist knowledge, perceptions and training opportunities regarding maternal-foetal medicine in Canada, Qatar and Uganda recorded knowledge assessment mean scores of 62.9, 53.3, and 57.7% respectively for each country (*p* < 0.05). The scores were interpreted as low, alluding to it being responsible for the creation of a knowledge gap in practice [20]. Similarly, a Pakistan study where maternal and child health care content was integrated in a medical curriculum showed that assessment mean scores of above 78% indicated that participants had acquired adequate knowledge and skills [22].

### Motivation for curriculum review and integration

This study reported on a maternal and child health care curriculum content that although it incorporated different types of learning activities (on campus theoretical and experiential) was largely not integrated. Indeed, traditional, discipline-based methods of teaching are usually done in silos and compartmentalises learning, thereby depriving the student of the opportunity to integrate curriculum content and acquire a holistic education that is applicable to real life situations. A nine-year study on the impact of changing from traditional to integrated problem-based learning at a school of pharmacy in South Africa showed that student pass rate increased after curriculum integration [46]. Zaman and Rauf explained the argument of medical educationalists that most health issues were transdisciplinary and countered the disciplinary approach used in most medical schools. They pointed out that if the curriculum was integrated students would be more likely to develop a preventive as opposed to a curative care mentality [22]. Indeed, focus should be on improving a preventative mentality in maternal and child health care as much as the obstetric causes [3].

Although no study of community pharmacists’ maternal and child health care knowledge, skills and attitude has been done in South Africa, some studies from around the world indicate a gap and recommend undergraduate curriculum review, continuing professional development courses for practicing pharmacists and access to maternal and child health care resources [18, 20, 47]. The call for undergraduate curriculum review indicates a deficiency in maternal and child health care curriculum content and lack of programme integration which may be attributed to pharmacy educators’: (1) lack of knowledge of the role of pharmacists in maternal and child health care, (2) lack of knowledge of the policy and guidelines that support service delivery, (3) lack of knowledge of local need for such services, and (4) non-involvement in solving the problem where one exists [22]. In South Africa, the issue is further complicated by the proliferation of big chain pharmacies that limit patient consultation time [48] and the attendant practice of nurse-pharmacist partnerships where nurses attend to all maternal and child health care issues within pharmacies like in the public sector. This practice confirms to pharmacy graduates and undergraduates alike the absence of a role for them in maternal and child health care [49]. This is contrary to global trends in developed countries where pharmacists are extending their roles in primary health care and practicing at the top of their licenced capabilities [13–15]. Social accountability and needs based education proponents recommend that undergraduate education must meet the needs of the population to be served [50]. This can only be achieved through properly designed and integrated curriculum content, skills practical and experiential learning programmes for pharmacy undergraduates in maternal and child health care like that of their medical counterparts [22].

### Limitations

The outcome of this study may not be generalisable since it was carried out at one pharmacy school in South Africa with a single cohort of participants. Additionally, it was difficult to find studies to compare these results with, because most knowledge studies would focus on specific aspects of either reproductive and sexual health, maternal and antenatal care, or neonatal and child care and not on all aspects. This reinforces the fragmented approach to maternal and child health care training which opposes the concept of a continuum of care.

The sample population for this study posed a limitation as students were generally unwilling to participate in assessments that did not contribute toward their academic grades, and one that would analyse and disseminate their scores even if anonymized. Since convenient sampling and not randomised or purposive sampling methods was used, with about 61% of the class participating in the study, the result may not be a true reflection of the class performance. Written evaluation of infant growth assessment skills rather than traditional objective structured clinical examination or practical evaluation due to lack of adequate man power may have compromised the result of that section.

## Conclusion

The study identified a knowledge and skills gap in maternal and child health care amongst fourth year pharmacy students especially in the skills acquisition to assess infant growth. Better outcomes were observed in maternal and antenatal care and neonatal and child care with the best outcome in reproductive and sexual health. This was thought to be due to the inadequacy of curriculum content exposure and fragmented teaching methods that participants were exposed to for maternal and child health care related topics. Based on the result of this study, it is recommended that maternal and antenatal care, and neonatal and child care topics should be allocated adequate lecture time slots and an on-campus infant skills assessment practical be introduced before the SLiP programme. All the components of maternal and child health care should be integrated to ensure optimal student learning and to produce pharmacy graduates that are fit for service.

## Supplementary Information

**Additional file 1.** Questionnaire used for data collection in the study

## Data Availability

The datasets used and/or analysed during the current study are available from the corresponding author on reasonable request.

## Abbreviations

EBF: Exclusive breastfeeding
HIV: Human immunodeficiency virus
IQR: Interquartile range
MAC: Maternal and antenatal care
MCH: Maternal and child health
MUAC: Mid upper arm circumference
NCC: Neonatal and child care
NHI: National Health Insurance
ORS: Oral rehydration solution
PHC: Primary health care
PRN: Practice and Research Network
RSH: Reproductive and sexual health
SD: Standard deviation
SDG: Sustainable development goals
SLiP: Service learning in pharmacy
USA: United States of America
WHO: World Health Organization
